# Complete genome sequence of *Parvimonas micra* strain JM503A, a genetically tractable dental abscess clinical isolate

**DOI:** 10.1128/mra.00315-24

**Published:** 2024-06-11

**Authors:** Dustin L. Higashi, Anh Nguyen, Hua Qin, Christina Borland, Elizabeth A. Palmer, Nicolas Biais, Jens Kreth, Justin Merritt

**Affiliations:** 1 Division of Biomaterial and Biomedical Sciences, Oregon Health and Science University, Portland, Oregon, USA; 2 College of Liberal Arts and Sciences, Portland State University, Portland, Oregon, USA; 3 Department of Pediatric Dentistry, Oregon Health and Science University, Portland, Oregon, USA; 4 Laboratoire Jean Perrin, Sorbonne Université, Paris, France; 5 Department of Molecular Microbiology and Immunology, Oregon Health and Science University, Portland, Oregon, USA; Rochester Institute of Technology, Rochester, New York, USA

**Keywords:** *Parvimonas micra*, genome

## Abstract

*Parvimonas micra* is a pathobiont of humans that is often found in abundance at sites of mucosal inflammation as well as within malignant tumors. Here, we report the complete genome sequence of *P. micra* strain JM503A, which is a genetically tractable clinical isolate derived from a human odontogenic abscess specimen.

## ANNOUNCEMENT

*Parvimonas micra* is a Gram-positive obligate anaerobe and a typical member of the human oral cavity and gastrointestinal tract (1, 2). *P. micra* is highly enriched at numerous sites of mucosal dysbiotic disease and is closely associated with multiple types of cancer (3, 4). *P. micra* strain JM503A, previously called A28 (5), was isolated from an odontogenic abscess of a patient undergoing a tooth extraction at Oregon Health and Science University Hospital. This strain exhibits a high level of natural competence, making it a suitable candidate for detailed genetic studies (5).

A stock of the original clinical isolate of *P. micra* strain JM503A was stored at −80°C in a supplemented brain heart infusion (sBHI) medium (BD, Sparks, MD) containing 25% glycerol. Bacteria were cultured on sBHI agar in anaerobic conditions at 37°C as described previously, and a single colony was harvested and expanded on fresh sBHI agar plates (6). JM503A agar cultures were harvested and transferred to liquid sBHI and maintained in anaerobic conditions at 37°C for 18 hours.

Genomic DNA (gDNA) was purified using a phenol-chloroform extraction procedure similar to that previously described (5) with the addition of a hexadecyltrimethylammonium bromide (CTAB) treatment (7). Bacteria were harvested from liquid sBHI cultures by centrifugation at 2,898 × *g* for 20 minutes. Following lysozyme, SDS, and proteinase K treatment, 5M NaCl was added to the DNA solution to a final concentration of 1.4 M followed by the addition of CTAB (11% final) and incubated for 10 minutes at 65°C. Phenol-chloroform extraction was performed as described previously with a final extraction using pure chloroform. The resulting DNA was further purified using the Genomic DNA Clean & Concentrator 10 prep kit (ZYMO RESEARCH, Irvine, CA) as per the manufacturer’s instructions.

DNA sequencing was performed at Plasmidsaurus (Eugene, OR) using the Oxford Nanopore Technologies platform (Oxford, UK). A long-read sequencing library was constructed using v14 library preparation chemistry (SQK-RBK114.96), and the barcoded samples were solid-phase reversible immobilization cleaned and pooled before adding the Rapid Sequencing Adapters to the tagged ends. Library sequencing was done using PromethION (R10.4.1 flow cell). Base calling was performed using the ont-doradod-for-promethion (v7.1.4) algorithm on super-accurate mode (N_50_ of 2,353 and 223,023 reads). A minimum raw read Qscore 10 was employed. The lowest 5% fastq reads were removed via Filtlong (v0.2.1) with option -p 95. Reads were downsampled to 250 Mb via Filtlong (using parameter -target bases 250,000,000), and a rough sketch of the assembly was created with Miniasm (v0.3) using -x ava-ont. Using information acquired from the Miniasm assembly, the reads were further downsampled to ~100× coverage with heavy weight applied to remove low-quality reads using Filtlong (mean q weight 10 –min length 300). Sequence assembly was performed using Flye (v2.9.1) with parameters selecting for high-quality ONT reads and polished using Medaka (v1.8.0) with basecall model r1041 e82 400bps sup v4.2.0. Contig analysis utilized Bandage (v0.8.1) run with option -color depth, and genome completeness and contamination were assessed using CheckM (v1.2.2) under default settings. Species identification was performed using Mash (v2.3) against RefSeq genomes and plasmids and Sourmash (v4.6.1) against GenBank.

The complete JM503A genome is 1,549,037 bp with a G + C content of 28% and a sequencing coverage depth of approximately 102×. Genome overlap was identified and trimmed using Flye (8), and the genome was rotated using Geneious Prime 2024.0.3 to put the start at *dnaA*. In total, 1,490 genes, including 42 tRNAs and 10 rRNAs, were annotated using the National Center for Biotechnology Information Prokaryotic Genome Annotation Pipeline (9–11).

## Data Availability

The genome sequence was deposited in NCBI under the accession no. CP148048.1. The raw sequencing data are available under BioProject PRJNA1088090 and in the Sequence Read Archive (SRA) SRR28357771.
